# *ASR* Enhances Environmental Stress Tolerance and Improves Grain Yield by Modulating Stomatal Closure in Rice

**DOI:** 10.3389/fpls.2019.01752

**Published:** 2020-02-14

**Authors:** Seong-Im Park, Jin-Ju Kim, Sun-Young Shin, Young-Saeng Kim, Ho-Sung Yoon

**Affiliations:** ^1^ Department of Biology, College of Natural Sciences, Kyungpook National University, Daegu, South Korea; ^2^ School of Life Sciences, BK21 Plus KNU Creative BioResearch Group, Kyungpook National University, Daegu, South Korea; ^3^ Research Institute for Dok-do and Ulleung-do, Kyungpook National University, Daegu, South Korea; ^4^ Advanced Bio-Resource Research Center, Kyungpook National University, Daegu, South Korea

**Keywords:** abscisic acid, abscisic acid-, stress-, and ripening-induced genes, grain yield, salt and drought stress tolerance, stomatal closure, transgenic rice plant

## Abstract

Abscisic acid-, stress-, and ripening-induced (*ASR*) genes are involved in responding to abiotic stresses, but their precise roles in enhancing grain yield under stress conditions remain to be determined. We cloned a rice (*Oryza sativa*) *ASR* gene, *OsASR1*, and characterized its function in rice plants. *OsASR1* expression was induced by abscisic acid (ABA), salt, and drought treatments. Transgenic rice plants overexpressing *OsASR1* displayed improved water regulation under salt and drought stresses, which was associated with osmolyte accumulation, improved modulation of stomatal closure, and reduced transpiration rates. *OsASR1*-overexpressing plants were hypersensitive to exogenous ABA and accumulated higher endogenous ABA levels under salt and drought stresses, indicating that *OsASR1* is a positive regulator of the ABA signaling pathway. The growth of *OsASR1*-overexpressing plants was superior to that of wild-type (WT) plants under paddy field conditions when irrigation was withheld, likely due to improved modulation of stomatal closure *via* modified ABA signaling. The transgenic plants had higher grain yields than WT plants for four consecutive generations. We conclude that *OsASR1* has a crucial role in ABA-mediated regulation of stomatal closure to conserve water under salt- and drought-stress conditions, and *OsASR1* overexpression can enhance salinity and drought tolerance, resulting in improved crop yields.

## Introduction

The continuous use of irrigation systems and increasingly frequent dry periods lead to the simultaneous occurrence of salinity and drought on arable land around the world (Hu and Schmidhalter, 2005). Both salinity and drought are significant plant stressors with major impacts on plant development and grain yield, causing economic losses in agricultural production (Flowers, 2004; Godfray et al., 2010). Much research is focused on improving agricultural production and developing stress-tolerant crops, but current efforts still fail to meet the demands of food security in the face of global population growth, global warming, water shortages, and soil salinization (Hu and Xiong, 2014).

Plants have evolved numerous mechanisms to adapt to salinity and drought, such as stomatal adjustment, osmoregulation, selective uptake, and compartmentation of ions (Blumwald, 2000). These physiological responses are primarily regulated by the plant stress hormone abscisic acid (ABA) (Verma et al., 2016). ABA accumulates rapidly in response to salinity and drought conditions and mediates numerous responses that help plants survive the stresses. ABA regulates guard cells to maintain their water content under osmotic stress and induces the expression of genes encoding proteins involved in cell dehydration resistance, including late embryogenesis abundant (LEA) proteins, water transporters, protein kinases, protein phosphatases, transcription factors, and enzymes involved in osmolyte synthesis (Fujita et al., 2011; Golldack et al., 2014).

ABA-, stress-, and ripening-induced (ASR) proteins were first reported in tomato (*Solanum lycopersicum* L.) (Iusem et al., 1993). These plant-specific, small, and hydrophilic proteins are induced by ABA during drought stress and ripening. Subsequently, *ASR* genes have been identified in various monocot (Riccardi et al., 1998; Vaidyanathan et al., 1999), dicot (Van Berkel et al., 1994; Hong et al., 2002), and gymnosperm (Shen et al., 2005) species. In rice, there are six *ASR* genes located in four different chromosomes. These genes are thought to arise from tandem and whole genome replication based on their location on the chromosome and gene sequence similarities (Philippe et al., 2010). The ASR protein structure consists of a short N-terminal consensus sequence comprising six to seven His residues that might constitute a Zn-binding site and a DNA-binding site containing about 70 amino acids with a highly conserved ABA/WDS domain (Cakir et al., 2003; Henry et al., 2011; Jia et al., 2016) in the C-terminal region.

Recent studies report that some *ASR* genes are located in the nucleus and encode transcription factors, regulating gene expression during stress responses (Cakir et al., 2003; Hu et al., 2013; Dominguez and Carrari, 2015). ASR proteins, such as the lily pollen ASR and the rice ASR5, have been localized in both nuclei and cytoplasm (Wang et al., 2005; Konrad and Bar-Zvi, 2008; Arenhart et al., 2013). These studies suggest that ASR proteins may affect transcriptional modulation. Although the exact physiological role of ASR proteins is unknown, they are presumed to have functional duality in plants. A previous study suggested that ASR proteins may serve as a downstream component of the signal transduction cascade involved in plant cell responses to various stressors (Maskin et al., 2001). ASR proteins also participate in mediating plant developmental processes, including senescence, fruit ripening, and pollen maturation (Dominguez and Carrari, 2015). Many *ASR* genes respond to ABA and abiotic stresses, such as drought, salt, cold, and limited light (Hong et al., 2002; Jeanneau et al., 2002; Yang et al., 2005; Liu et al., 2010; Hu et al., 2013; Golan et al., 2014; Wang et al., 2016b). The role of ASR proteins in drought-stress tolerance was first reported in lily through the *LLA23* gene, which encodes an ASR protein. Overexpression of lily *ASR* conferred drought and salt tolerance to *Arabidopsis thaliana* by increasing the expression of ABA/stress-regulated genes (Yang et al., 2005). Transgenic tobacco plants overexpressing the *ASR* gene of brome grass (*Brachypodium distachyon*, *BdASR1*) or wheat (*Triticum aestivum*, *TaASR1*) exhibited improved tolerance to drought stress (Hu et al., 2013; Wang et al., 2016b). Recombinant *Escherichia coli* cells expressing the *ZnAsr1* gene from *Ziziphus nummularia* displayed enhanced tolerance to drought stress induced by polyethylene glycol (PEG) (Padaria et al., 2016). Transgenic rice plants overexpressing *OsASR1* or *OsASR3* display enhanced photosynthetic efficiency under cold and drought stress compared to controls (Joo et al., 2013). Although *ASR* genes were first identified more than 20 years ago and respond to a variety of abiotic stresses, a complete map of their molecular and physiological functions under salinity and drought stress is still lacking. The majority of studies on *ASR* genes were performed under controlled conditions in laboratories and greenhouses. There are few reports on the role of *ASR* genes in salinity and drought tolerance or crop yield in natural fields, although research using these approaches is in progress (Qin et al., 2016).

This study builds on our previous work wherein we found that the *ASR1* gene of rice confers stress resistance to yeast cells by promoting scavenging of reactive-oxygen species (ROS) and performing chaperone-like activities (Kim et al., 2012). Here, we report on the role of the rice *OsASR1* gene in salinity and drought tolerance and crop grain yield in laboratory and natural paddy field conditions using *OsASR1*-overexpressing rice plants. Our results show that rice *OsASR1* is a promising candidate for transgenic breeding of plants with enhanced resistance to both salinity and drought, thereby reducing the requirements for irrigation and improving crop yield.

## Materials and Methods

### Plasmid Construction and *Agrobacterium*-Mediated Rice Transformation

Total RNA was extracted from *Oryza sativa* L. *japonica* (*O. sativa*) leaves using an RNeasy Plant Mini Kit (Qiagen, Valencia, CA, USA), and then cDNA was synthesized using the SuperScript III First-Strand Synthesis System (Life Technologies). The regions encoding the *OsASR1* cDNA (GenBank accession no. AK062319) were amplified by PCR using Ex Taq polymerase (TaKaRa, Tokyo, Japan) with a specific primer set (OsASR1-FC and OsASR1-RC; **Supplementary Table S2**). The amplified regions were cloned into the *pENTR/SD/D-TOPO* vector (Invitrogen, Carlsbad, CA, USA), and then the LR recombination reaction was performed with a *pGA2897* binary vector modified from *pGA1611*.

The plasmid harboring the cloned gene under control of the maize ubiquitin promoter was introduced into *Agrobacterium* strain LBA 4404 and used to transform rice calli produced from scutella of mature seeds of *Oryza sativa* cv. Ilmi. The method used for *Agrobacterium*-mediated rice transformation is described below and was based on that of Hiei et al., 1994. A. *tumefaciens* strain LBA4404 was grown for 3 d on Agrobacterium minimal (AB) medium containing 50 mg·L^-1^ hygromycin. Cells were collected by centrifugation at 2,560 rpm for 30 min and resuspended in AAM medium at a density of 3–5 × 10^9^ cells·mL^-1^. The rice calli described above were immersed in this bacterial suspension for 10–20 min and incubated at 25°C in the dark for 3 d. After co-cultivation, the materials were washed four times with 250 mg·L^-1^ cefotaxime in sterile water and cultured in 2N6-CH medium for 3 weeks. A white callus proliferated in the first selection medium. The callus was cultured on N6-7-CH medium for 10 d and incubated in a regeneration medium (N6S3-CH) at 25°C under continuous illumination (1500 lux). Regenerated plants were eventually transferred to soil in pots and grown in a greenhouse maintained at 30 ± 4°C during the day and 24 ± 2°C during the night with relative humidity of 60–70%.

Approximately 20 independent T_0_ transgenic rice plants were screened using hygromycin. Among the transformants, 16 independent T_1_ transgenic plants grown from T_1_ seeds were selected using hygromycin and PCR genotyping. Four independent, homozygous T_2_ plants were identified using the same process, which we refer to as TR1, TR2, TR3, and TR4.

### Identification of the *OsGS* Transgene That Was Inserted Into Rice

We used the adapter ligation PCR method to isolate flanking sequences of T-DNA. First, 0.5 µg genomic DNA were digested with 2 U of HpaII restriction enzyme and ligated with 5 U of T4 DNA ligase (Takara, Shiga, Japan) in a 20-µL reaction volume. The reaction mixture, containing T4 DNA ligase buffer and 50 pmol of the adapters, was incubated at 37°C for 1 h. The first PCR was conducted in 20 µL reaction volume containing PCR pre-mix, 0.5 pmol each primer (Ada1 and LB1 or RB), and 1 µL digestion or ligation product. PCR was performed in a PTC-200 thermal cycler (MJ Research, Watertown, MA, USA) and conducted using the primers Ada2 and LB2 or RB2 as follows: first denaturation step at 95°C for 5 min, 20 cycles of 30 s at 94°C and 1 min at 72°C, followed by a final elongation step at 72°C for 10 min. The second PCR was conducted with 5 µL of the first PCR product under the conditions of first denaturation step at 94°C for 5 min, 40 cycles of 30 s at 94°C, 30 s at 60°C, and 1 min at 72°C, followed by a final elongation step at 72°C for 10 min. Amplified products were loaded onto a 1% agarose gel. The PCR products were purified using the HiYield™ Gel/PCR DNA Extraction Kit (Real Biotech Corporation, Taiwan) and sequenced by ABI3730XL (Applied Biosystems, Foster City, CA, USA) using LB2 or RB2 primer (Lim et al., 2016).

### Plant Materials and Stress Treatments

Wild-type rice seeds or seeds from the transgenic plants were germinated for 6 d in a growth chamber at 28°C after which the seedlings were transplanted into nursery bed soil (Pungnong, Seoul, South Korea) in plastic pots (10 cm diameter, 9 cm height) at 85% soil field capacity and grown in a greenhouse maintained at 30 ± 4°C during the day and 24 ± 2°C during the night with relative humidity of 60–70% until use. Leaves, roots, and stems were sampled at vegetative and reproductive stages for analysis of *OsASR1* expression. The reproductive stage was identified as the first appearance of the inflorescence meristem. Rice embryo samples were isolated from mature seeds.

The stress treatment was conducted in greenhouse conditions maintained at 30 ± 4°C during the day and 24 ± 2°C during the night with relative humidity of 60–70% and average 1200 µmol m^−2^ s^−1^ photosynthetic photon ﬂux density (PPFD) in July (15-h light/9-h dark cycles) until use. The PPFD in greenhouse was measured at 12pm. Expression of *OsASR1* was analyzed in four-week-old plants exposed to several types of stress. ABA and salt stress were imposed by soaking the plants grown in pots with soil in 100 μM ABA or 200 mM NaCl solution, respectively. Drought stress was imposed by withholding water. Leaves and roots were sampled at 0, 1, 3, 6, 12, or 24 h after imposition of stress.

For salt stress phenotype analysis, four-week-old *OsASR1*-overexpressing transgenic and WT plants transplanted into soil in pots were soaked in 200 mM NaCl solution for 7 d, and then allowed to recover for 5 d. The number of surviving plants were counted for each line. The drought-stress phenotype was assessed in four-week-old plants treated as follows: plants were not watered for 3 d. After 3 days of exposure to water-free conditions, the soil capacity was 35 ± 5%, and then plants were recovered with daily watering for the next 7 days. The survival percentage was then assessed.

All tests were repeated at least three times.

### qRT-PCR

**Figure 1:**
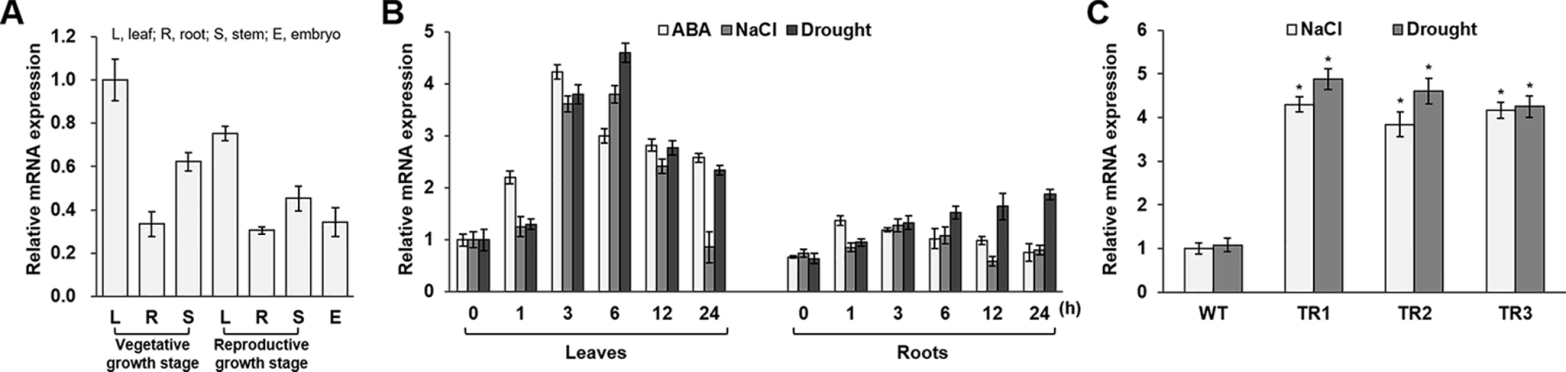
Expression analysis of the *OsASR1* gene and generation of *OsASR1*-overexpressing transgenic rice plants. **(A)** qRT-PCR analysis of expression of *OsASR1* levels in different organs of WT plants. *OsASR1* expression level in leaves of vegetative growth stage was considered to be 1; described as relative values. **(B)** Stress-inducible expression of *OsASR1* under ABA, NaCl, and drought stress in leaves and roots of WT rice plants. *OsASR1* expression difference was calculated relative to the expression level in leaves of untreated WT plants. **(C)** qRT-PCR analysis of *OsASR1* expression in transgenic and WT plants exposed to 200 mM NaCl and drought for 6 h. *OsASR1* expression difference was calculated relative to the expression level in WT leaves. Error bars indicate standard error (SE) based on three replicates. TR, *OsASR1*-overexpressing transgenic rice plants; WT, wild-type rice plants. Asterisks indicate significant differences between treatments determined by Student's *t*-test (*P* < 0.05).

Total RNA was extracted using TRIzol reagent (Takara) according to the manufacturer's instructions, and reverse transcription reactions were performed with 2 μg total RNA using the SuperScript III First-Strand Synthesis System. Expression of genes after various treatments in rice were analyzed by qRT-PCR. The qRT-PCR analysis was performed using the TB Green Premix Ex Taq II (Tli RNaseH Plus) Kit (TaKaRa) and QuantStudio3 Real-Time PCR system (Applied Biosystems, Foster City, CA, USA). The 20-μl reactions contained 12.5 μl of TB Green Premix Ex Taq II (Tli RNaseH Plus), 0.4 μl of ROX Reference Dye II, 200 ng of cDNA template, 0.4 μl of each primer (10 μM), and 6.26 μl of PCR‐grade water. All samples were analyzed in biological triplicates as follows: initial preheating at 95°C for 30 s, followed by 40 cycles of 95°C for 3 s and 60°C for 30 s. The rice tubulin (*Tub*) gene was used as a positive control. Fold changes in mRNA were calculated relative to the calibrator. The level of *OsASR1* expression in **Figure 1** is presented relative to that in WT leaves (which were assigned an expression level of 1) under normal condition. Normalization and relative quantification were conducted using the 2^-∆∆Ct^ method (Livak and Schmittgen, 2001). The primers used for qRT-PCR analysis are listed in the **Supplementary Table S2**.

### Analysis of Relative Water Content (RWC), MDA, Proline, and Total Soluble Sugars

To determine RWC under salt and drought-stress conditions, the midsection of the third fully expanded leaf blade of *OsASR1*-overexpressing transgenic and WT plants was sampled before, during, and after treatment with 200 mM salt and drought stress for 7 and 3 d, respectively. After measuring the fresh weight (FW) of the rice leaves, the leaves were soaked in distilled water for 4 h at room temperature under constant light, and turgid weight (TW) was measured. The leaves were then dried for 24 h at 80°C to determine the total dry weight (DW). RWC was calculated according to the formula RWC = (FW–DW)/(TW–DW)×100 (Gao et al., 2015). The amount of malondialdehyde (MDA) was determined by the thiobarbituric acid (TBA)-based colorimetric method (Hodges et al., 1999). Briefly, 0.1 g leaf sample was homogenized using liquid nitrogen, mixed with a solution containing 20% (w/v) trichloroacetate and 0.65% (w/v) thiobarbituric acid, heated in a water bath at 95°C for 25 min, and centrifuged for 10 min at 12,000 rpm at 4°C. Finally, the MDA concentration of the resulting supernatant was measured at 532 nm based on 600 nm and calculated using a molar extinction coefficient of 1.56 × 10^5^ cm^-1^ M^-1^ for MDA.

For analysis of free proline and total soluble sugars, the second leaves were sampled and ground. Then, 0.6 mL of 80% (v/v) hot ethanol was added, and the sample was mixed for 2 min. After centrifugation at 1200 rpm for 5 min, the supernatant was collected. The pellet was re-extracted twice with 0.4 mL of 80% ethanol, then all supernatants were combined. The content of free proline dissolved in ethanol was measured using the ninhydrin method (Wren and Wiggall, 1965). The total soluble sugars dissolved in ethanol were determined using the anthrone method (Irigoyen et al., 1992). Fifty-microliter samples dissolved in ethanol were dried under vacuum, dissolved in 0.1 mL de-ionized water, and proteins were removed by mixing with 0.1 mL 0.3 N Ba(OH)_2_ and 0.1 mL 5% ZnSO_4_. After centrifugation at 12000 rpm for 5 min, 0.1 mL of supernatant was reacted with 0.4 mL freshly prepared anthrone reagent (100 mg anthrone in 50 mL 95% H_2_SO_4_) at 100°C for 10 min and cooled at 4°C. Finally, the total soluble sugars were determined by measuring absorbance at 620 nm in a spectrophotometer, using glucose as the standard.

### Histochemical Staining

Four leaves from each transformant and WT plant were harvested 7 weeks after they had been transplanted in paddy fields. Leaves were cut and soaked in 0.1% nitro blue tetrazolium (NBT) in 10 mM sodium phosphate buffer for detection of superoxide ion (O_2_^-^) (Lin et al., 2009). Then, the samples were incubated in a growth chamber for 48 h and the chlorophyll was removed with ethanol (95%). The superoxide anion stains dark blue (Awasthi et al., 2018).

### Analysis of Inorganic Solutes

Four-week-old plants were treated with 200 mM NaCl solution for 3 d and the Na^+^ concentration of leaves was measured. Total leaves from five plants of each transformant and WT plant were homogenized using a mortar and pestle in liquid nitrogen. The ground powder was placed in a 50-mL tube and dried in an oven at 80°C for 72 h. Dried samples (50 mg) were heated for 1 h at 95°C in distilled water, and then filtered through polyimide syringe filters. Na^+^ and K^+^ ion concentrations (calculated as NaCl equivalents) were determined using inductively coupled plasma optical emission spectroscopy (PerkinElmer, Shelton, CT, USA).

### Imaging Stomatal Opening and Measuring Stomatal Conductance, Transpiration Rate, and Net Photosynthetic Rate

Leaves of *OsASR1*-overexpressing transgenic and WT plants exposed in the greenhouse to 200 mM NaCl or drought (5 and 2 d, respectively) or grown in paddy fields without irrigation for 4 d were directly fixed with formaldehyde-acetic acid alcohol (FAA) solution containing 5 mL of ethanol, 0.5 mL of acetic acid, 1 mL of 37% formaldehyde, and 3.5 mL of distilled water. Samples were incubated under vacuum (550 mmHg) and then transferred to a fresh FAA solution at 4°C overnight. Samples were then washed three times with sodium phosphate buffer, dehydrated through an ethanol series at room temperature, and stored in 100% ethanol before use. The stomatal images were obtained with a scanning electron microscope (SEM) (SU 8220, Hitachi, Tokyo, Japan), and the percentages of stomata that were completely open, partially open, and completely closed were calculated (*n =* 100 stomata). A portable CO_2_ gas analyzer (LCi, ADC BioScientific Ltd.) was used to test the stomatal conductance, transpiration rate, and net photosynthetic rate in transgenic and WT plants exposed in the greenhouse to 200 mM NaCl or drought for 5 and 2 d, respectively. Net photosynthetic rate, stomatal conductance, and transpiration rate were measured in greenhouse conditions maintained at 30 ± 4°C during the day with relative humidity of 60–70% at 2–3 pm in July. At the time of measurement, the PPFD in the greenhouse was about 843–867 µmol m^−2^ s^−1^. Second leaves of four-week-old rice plants from each line were measured.

### Endogenous ABA Contents Measurement and Exogenous ABA Sensitivity Assay

The endogenous ABA levels were determined by a Shimadzu Prominence HPLC System (Shimadzu Scientific Instruments, Inc., Columbia, MD, USA) (Changan et al., 2018). Second leaves (2 g) from each line were sampled and put into liquid nitrogen, ground into powder, and extracted into 5 mL 98% (v/v) acetone for 12 h at 4°C. The supernatant was filtered using Whatman No. 1 filter paper. The filtrate was evaporated with evaporator (IKA RV 10 rotary evaporator; IKA^®^—Werke GmbH & Co., Germany) to remove the acetone. The soluble material deposited on the walls of the glass tube was dissolved in 1% (v/v) acetic acid, and the samples were filtered through a sterilized Millipore filter and injected into the HPLC system. Standard solutions of ABA were directly prepared in 95% (v/v) methanol. Separation was carried out on a Waters Symmetry C18 column (4.6×250 mm, 5 µm) and maintained at 30°C with a gradient elution at the flow rate of 1 mL·min^−1^. A 1% acetic acid-methanol (40:60, v/v) was the mobile phase. The target component was quantified by the peak areas at the maximum wavelength of 260 nm. The run time was 20 min.

To test the sensitivity to ABA at the germination stage, 30 seeds were placed in Petri dishes containing 0, 2.5, and 5 μM ABA at 16-h light/8-h dark cycles (28°C), and the number of seeds germinated on the fifth day was counted. The seeds of *OsASR1*-overexpressing transgenic and WT plants germinated on normal 1/2 MS medium were transplanted to 1/2 MS medium containing 0, 2.5, and 5 μM ABA at 16-h light/8-h dark cycles (28°C). The shoot and root lengths of each seedling were measured after 10 d.

### Evaluation of Drought Tolerance in Paddy Fields

To generate drought conditions in paddy fields, the fields were maintained by watering using an irrigation system for 3 d followed by 4 d of withholding water beginning at 4 weeks after transplantation into the field. Once the plants were under drought conditions, we counted the numbers of tillers on rice plants at 4–7 weeks after transplantation into the paddy field to measure stress tolerance in an intermittently irrigated environment.

### Agronomic Trait Analysis

Four-week-old seedlings of *OsASR1*-overexpressing transgenic and WT plants were transplanted into a paddy field located at the Gunwi campus of Kyungpook National University each growing season from 2015 (T_3_) to 2018 (T_6_). The paddy field was fertilized once a year using commercially available fertilizer, Powerplus (FarmHannong, Seoul, South Korea), based on the fertilization reference value. A completely randomized block design was employed, and each plot contained 30 plants with two rows for each line, 0.2 m between plants, and 0.3 m between plots. When the transgenic plants reached maturity, agronomic traits were calculated for 10 plants per plot. They were harvested and threshed by hand. Filled grains were randomly counted and weighed. The following agronomic traits were scored: total plant weight (g), culm weight (g), root weight (g), number of tillers per plant, number of panicles per plant, total grain yield (g), number of grains per panicle, number of spikelets per panicle, and 1000-grain weight (g).

### Identification of Climatic Conditions in the Paddy Field

Temperature and precipitation data for the paddy field located at the Gunwi campus of Kyungpook National University were obtained from the Korea Meteorological Administration (http://www.kma.go.kr/index.jsp).

### Statistical Analysis

For the biochemical measurements (RWC, MDA, proline, total soluble sugars, Na^+^, K^+^, and ABA) in rice plants, each sample contained three replicates, and each replicate contained five seedlings. For the germination test of in rice seeds on wet filter paper containing 0, 2.5, or 5 μM ABA, each sample contained three replicates, and each replicate contained 30 seeds. In order to measure the tiller number of rice plants at 4–7 weeks after transplantation into the paddy field, we counted the numbers of tillers on the same ten plants per each transgenic and WT plants. Comparisons between individual data points were performed using Student's *t*-test with *P* < 0.05 considered as significant. All experiments were performed at least in triplicate, and all results are expressed as the mean ± SE.

### Accession Numbers

Genes and their associated accession numbers from the GenBank database are as follows: *Tubulin* (AK072502), *OsASR1* (AK062319), *OsNCED4* (AY838900), *OsNCED5* (AY838901), *OsRab16C* (AK071366), and *OsRab16D* (AK109096). Genes and their associated accession numbers from the Rice Annotation Project Database are as follows: *OsZEP1* (*LOC_Os04g37619*), *OsRab21* (*LOC_Os11g26790*), *OsMOC*1 (*LOC_Os06g40780*), *OsDLT* (*LOC_Os06g03710*), *OsMPH1* (*LOC_Os06g45890*), and *OsPROG1* (*LOC_Os01g03840*).

## Results

### Isolation of *OsASR1* and Generation of Transgenic Rice Plants Overexpressing *OsASR1*

To determine the tissue specificity of endogenous *OsASR1* expression in rice, we performed qRT-PCR analysis using mRNA from different rice tissues. *OsASR1* was expressed in all tested tissues, including leaf, root, stem, and embryo, regardless of developmental stage (**Figure 1A**). *OsASR1* expression in leaves was higher than in other tissues. To investigate *OsASR1* responses to abiotic stress, *OsASR1* transcript levels were analyzed in leaf and root tissues before and after exposure to ABA, NaCl, and drought (**Figure 1B**). The predominant expression of *OsASR1* in leaves was confirmed under all tested conditions. *OsASR1* transcripts in leaves were most strongly expressed at 3 h after ABA treatment and at 6 h after NaCl- and drought-stress treatment, whereas *OsASR1* was expressed continuously in root tissues under drought stress. These results suggest that *OsASR1* is responsive to abiotic stresses.

To further analyze the role of *OsASR1* in rice plants under salinity and drought conditions, we generated a construct for *Agrobacterium*-mediated rice transformation (Christensen and Quail, 1996) (**Supplementary Figure S1A**), which expressed the *OsASR1* coding sequence under the control of the constitutive maize ubiquitin promoter (*Ubi*). Four independent *OsASR1*-overexpressing transgenic lines were obtained (TR1, TR2, TR3, and TR4) and confirmed by genotyping (**Supplementary Figure S1B**). Next, to examine whether *OsASR1* was expressed under the regulation of the *Ubi* promoter at the transcriptional level, real-time PCR with OsASR1-F1 and OsASR1-R1 primers (**Supplementary Table S2**) was performed. We assessed expression in the leaves of four independent 4-week-old transgenic and WT rice plants grown in the greenhouse. *OsASR1* expression levels were about 5.9-fold greater in the TR1, TR2, and TR3 transgenic lines and 2-fold greater in the TR4 line than in WT rice plants (**Supplementary Figure S2**). We selected the three independent transgenic lines TR1, TR2, and TR3 based on their relatively high and similar gene expression levels for subsequent experiments.

A single copy of the *Ubi*::*OsASR1* transgene construct was inserted into the rice genome of the TR1 and TR2 lines (**Supplementary Table S1**). Two copies of the *Ubi*::*OsASR1* transgene construct were inserted into the rice genome of the TR3 line. T_1_ to T_6_ seeds were harvested from each transgenic plant line, and three homozygous T_6_ lines were used for further analysis of each line.

To evaluate the responses of the transgenic plants to salt and drought stress, the *OsASR1* expression levels were analyzed in 4-week-old transgenic plants treated with 200 mM NaCl and drought for 6 h (**Figure 1C**). The results clearly showed that *OsASR1* expression levels were upregulated in transgenic plants compared to wild-type (WT) plants.

### *OsASR1*Overexpression Significantly Enhances Salt and Drought Tolerance in Rice

**Figure 2:**
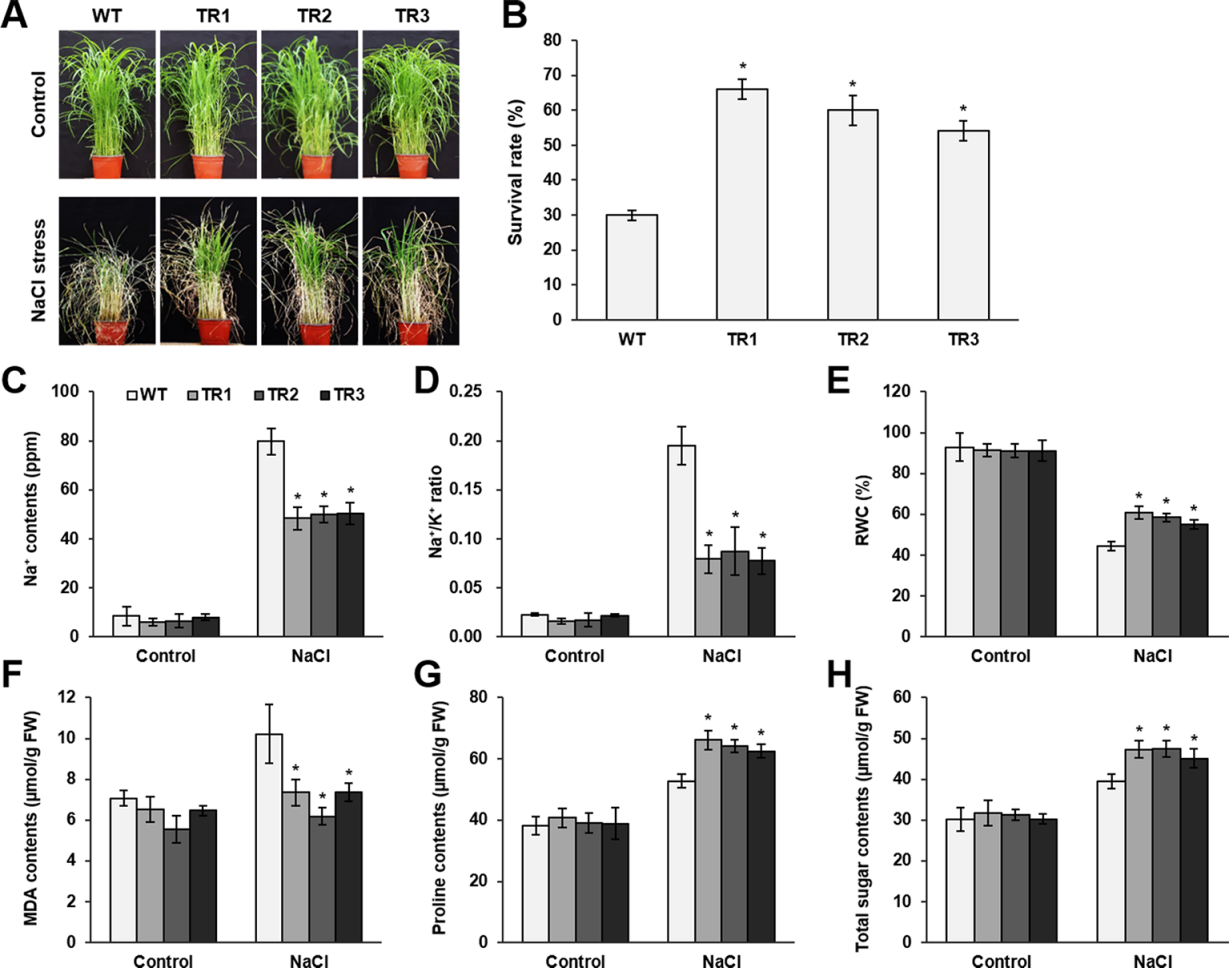
Analysis of salt tolerance in *OsASR1*-overexpressing transgenic plants. **(A)** Four-week-old seedlings (*n =* 50) after treatment with 200 mM NaCl for 7 d followed by recovery for 5 d. **(B)** Survival rates of transgenic and WT plants after recovery for 5 d. **(C)** Na^+^ contents and **(D)** Na^+^/K^+^ ratio of transgenic and WT plants. **(E)** Relative water content (RWC) of transgenic and WT plants after NaCl treatment for 7 d. **(F)** MDA levels, **(G)** proline contents, and **(H)** total soluble sugar contents in transgenic and WT plants. Analyses of Na^+^, Na^+^/K^+^ ratio, MDA, proline, and total sugar were carried out with leaves of rice plants treated with 200 mM NaCl for 3 d. Asterisks indicate significant differences between treatments determined by Student's *t*-test (*P* < 0.05).

**Figure 3:**
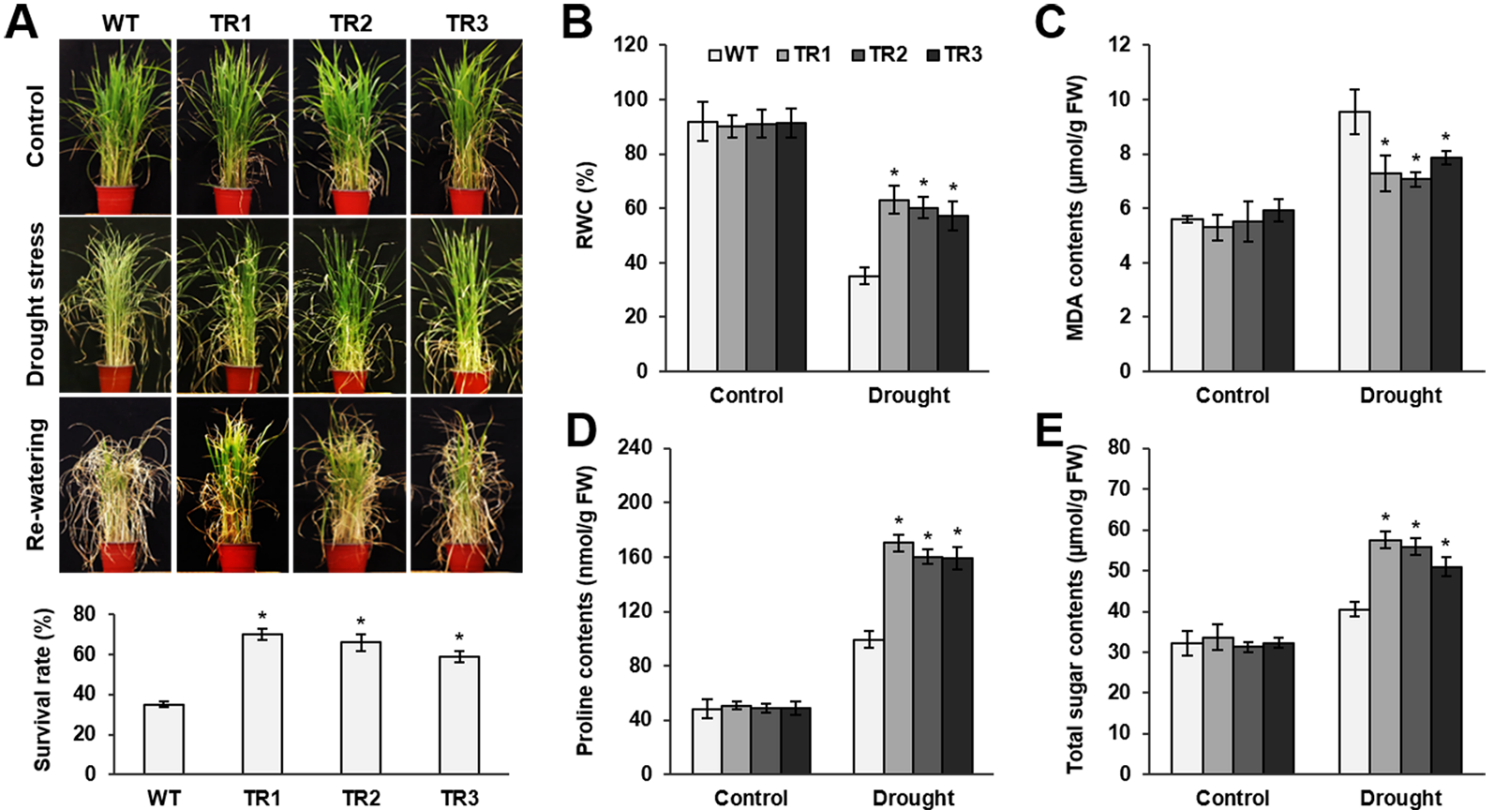
Analysis of drought tolerance in *OsASR1*-overexpressing transgenic plants. **(A)** Four-week-old seedlings (*n =* 50) after drought exposure for 3 d followed by recovery for 7 d (top). Survival rates of transgenic and WT plants after drought exposure for 3 d and recovery for 7 d (bottom). **(B)** Relative water content (RWC) after drought treatment for 3 d, **(C)** MDA content, **(D)** proline content, and **(E)** total sugar content in transgenic and WT plants. Analyses of MDA, proline, and total soluble sugars were carried out in rice plants subject to drought for 2 d. Asterisks indicate significant differences between treatments determined by Student's *t*-test (*P* < 0.05).

To perform a salt and drought tolerance test of *OsASR1*-overexpressing transgenic plants in soil, 4-week-old transgenic and WT plants at the seedling stage were exposed to 200 mM NaCl and drought for several days. Before stress treatment, no morphological differences were observed between the transgenic and WT plants (**Figures 2A** and **3A**, upper panel, **Supplementary Figure S3**). Stress-induced phenotypic changes, such as leaf rolling and wilting, occurred earlier in WT plants than in *OsASR1*-overexpressing plants (**Figures 2A** and **3A**, upper panel, **Supplementary Figure S3**). Because the phenotype of most of the WT plants was more affected by stress than the *OsASR1*-overexpressing plants after 3 and 7 d of drought and salt stress, respectively, these periods were used in further experiments (**Supplementary Figure S3**). The survival rate of transgenic plants was approximately 2-fold higher than that of WT plants (**Figures 2B** and **3A**, lower panel). The enhanced salt and drought tolerance in *OsASR1*-overexpressing plants prompted us to investigate physiological differences between the transgenic and WT plants. Therefore, we conducted ionomic analyses using leaves of transgenic and WT plants before and after exposure to 200 mM NaCl for 3 d. The Na^+^ contents and Na^+^/K^+^ ratios are known indicators of salt tolerance in plants under salt stress (Gao et al., 2003). Before NaCl treatment, the Na^+^ contents of both transgenic and WT plants were approximately 10 ppm (**Figure 2C**), with no significant differences between the values of each line. The Na^+^ content in WT plants after 3 d of salt stress was more than 1.6-fold higher than that of *OsASR1*-overexpressing plants (**Figure 2C**), and the Na^+^/K^+^ ratio of WT plants was 2.3-fold higher than that in transgenic plants (**Figure 2D**). The relative water content (RWC) is an indicator of osmotic stress tolerance. We measured RWC at 7 and 3 d under NaCl and drought stress in whole plants of transgenic and WT lines. *OsASR1*-overexpressing plants displayed enhanced survival under salt and drought stress compared to WT plants. The RWC of transgenic plants was approximately 1.5- and 2.0-fold higher than that of WT plants under NaCl and drought stress, respectively (**Figures 2E** and **3B**), suggesting that *OsASR1* may have a role in preventing water loss under salt- and drought-stress conditions. Malondialdehyde (MDA) is an indicator of membrane damage and was significantly lower in *OsASR1*-overexpressing plants than in WT plants under the same conditions (**Figures 2F** and **3C**).

To investigate the relationship between *OsASR1* expression and osmolyte synthesis, we measured the contents of free proline and total soluble sugars in *OsASR1*-overexpressing and WT plants subjected to salt and drought stress. The contents of free proline and water-soluble sugars increased in both *OsASR1*-overexpressing and WT plants after treatment with salt and drought, although the magnitude of increase significantly differed in the two plant lines (**Figures 2G, H** and **3D, E**). The results show that *OsASR1*-overexpressing plants accumulate higher contents of osmolytes such as proline and soluble sugars than WT plants, suggesting that *OsASR1*-overexpressing plants have enhanced tolerance to salt and drought stress compared to WT plants.

### *OsASR1* Is Involved in ABA-Mediated Stomatal Closure

**Figure 4:**
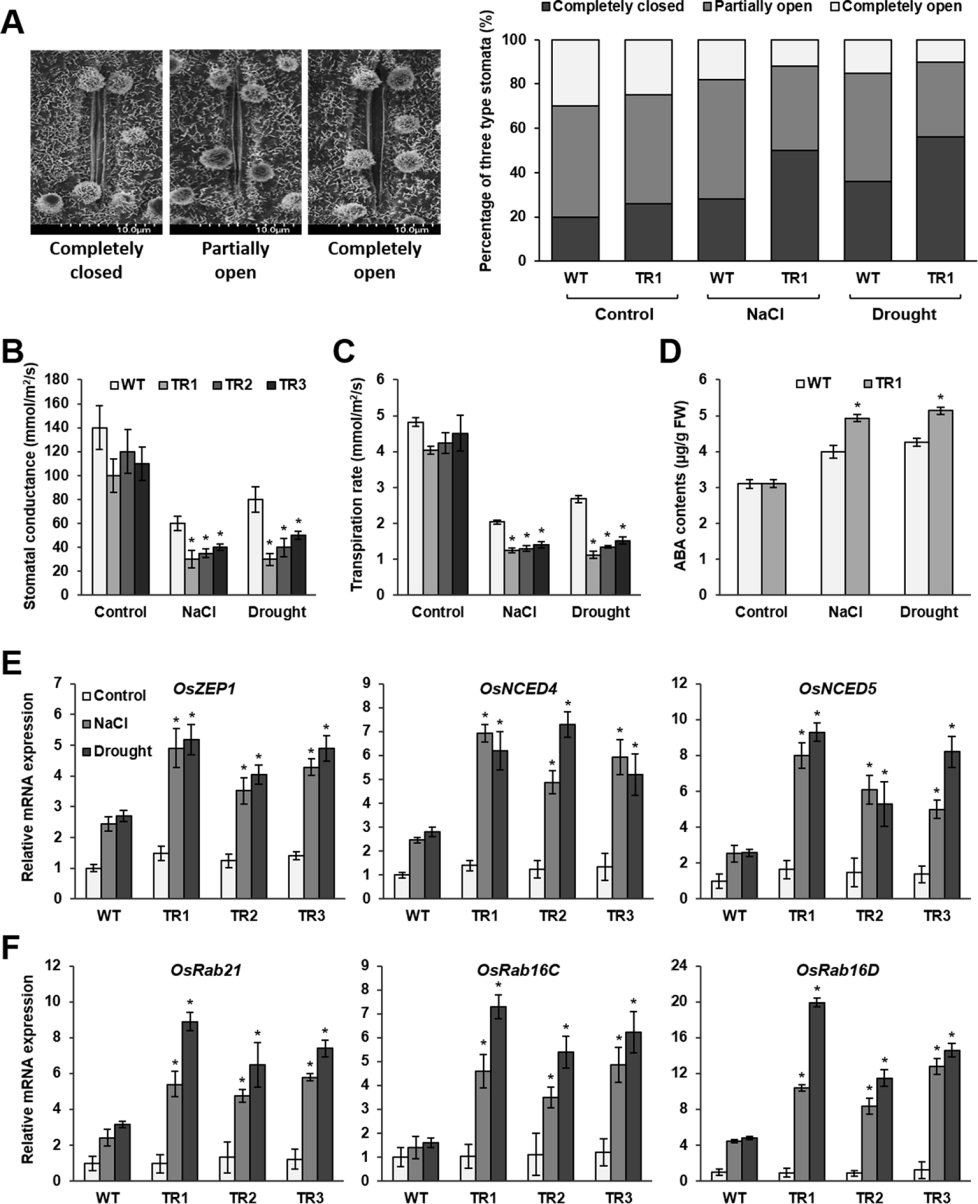
Stomatal closure, ABA accumulation, and expression of ABA biosynthesis genes and ABA-responsive genes in response to NaCl and drought stress in *OsASR1*-overexpressing transgenic plants. **(A)** Scanning electron microscopy images and the percentages of open, closed, and partially open stomatal apertures in leaves of transgenic and WT plants (*n =* 100 stomata). **(B)** Stomatal conductance in transgenic and WT plants (*n =* 5). **(C)** Transpiration rate in transgenic and WT plants (*n =* 5). **(D)** Endogenous ABA contents of transgenic and WT plants (*n =* 5). qRT-PCR analysis of the expression of **(E)** ABA biosynthesis genes and **(F)** ABA-responsive genes in transgenic and WT plants (*n =* 5) after salt (3 d) and drought (1 d) stress. Stomatal image, stomatal conductance, transpiration rate, and endogenous ABA content analyses were performed with rice leaves subjected to drought and salt stress for 2 and 5 d, respectively. Asterisks indicate significant differences between treatments determined by Student's *t*-test (*P* < 0.05).

Osmotic stress in plants induces water loss primarily through stomata. Therefore, we examined stomatal apertures in *OsASR1*-overexpressing transgenic plants, which had lower water loss than WT plants under salt and drought stress, using scanning electron microscopy (SEM). Under normal conditions, the percentages of completely closed, partially open, and completely open stomata were essentially the same in *OsASR1*-overexpressing and WT plants (**Figure 4A**). By contrast, the percentages of completely closed stomata were higher in *OsASR1*-overexpressing plants than in WT plants, and the percentages of completely open stomata were lower in *OsASR1*-overexpressing plants under salt and drought stress. In both transgenic and WT plants, the percentages of completely closed stomata were higher under drought stress than under salt stress. Furthermore, the stomatal conductance transpiration rates, and net photosynthetic rates were significantly reduced in *OsASR1*-overexpressing plants under salt and drought stress (**Figures 4B, C**, **Supplementary Figure S4**). These results suggest that the low percentages of open stomatal apertures and the high percentages of completely closed stomata in *OsASR1*-overexpressing plants reduced stomatal conductance and transpiration rate, thereby preserving endogenous water content and enhancing salt and drought tolerance by preventing saline absorption in rice.

ABA regulates stomatal movement. Therefore, we measured ABA contents in leaves of *OsASR1*-overexpressing and WT plants subjected to 200 mM NaCl and drought for 5 and 2 d, respectively. The results showed that ABA levels in transgenic plants were more than 1.25-fold higher than in WT plants (**Figure 4D**). We hypothesized that *OsASR1* had a major regulatory role in the ABA biosynthetic pathway and likely participated in an ABA‐dependent stress‐signaling pathway in response to salt and drought stress.

To test these hypotheses, we analyzed the transcript levels of ABA biosynthetic genes (*OsZEP1*, *OsNCED4*, and *OsNCED5*) and ABA-responsive genes (*OsRab21*, *OsRab16C*, and *OsRab16D*) in *OsASR1*-overexpressing and WT plants subjected to 200 mM NaCl and drought for 3 and 1 d, respectively. The results showed that the expression levels of all six genes were more strongly upregulated by both salt and drought stresses in *OsASR1*-overexpressing plants than in WT plants (**Figures 4E, F**), whereas the expression levels of these genes did not significantly differ in transgenic and WT plants under normal conditions. These combined results indicate that *OsASR1* has a significant role in the ABA‐dependent stress‐signaling pathway by upregulating ABA biosynthesis and the expression of ABA-responsive genes under abiotic stress conditions.

### *OsASR1* Overexpression Increases the Sensitivity to Exogenous ABA in Rice

**Figure 5:**
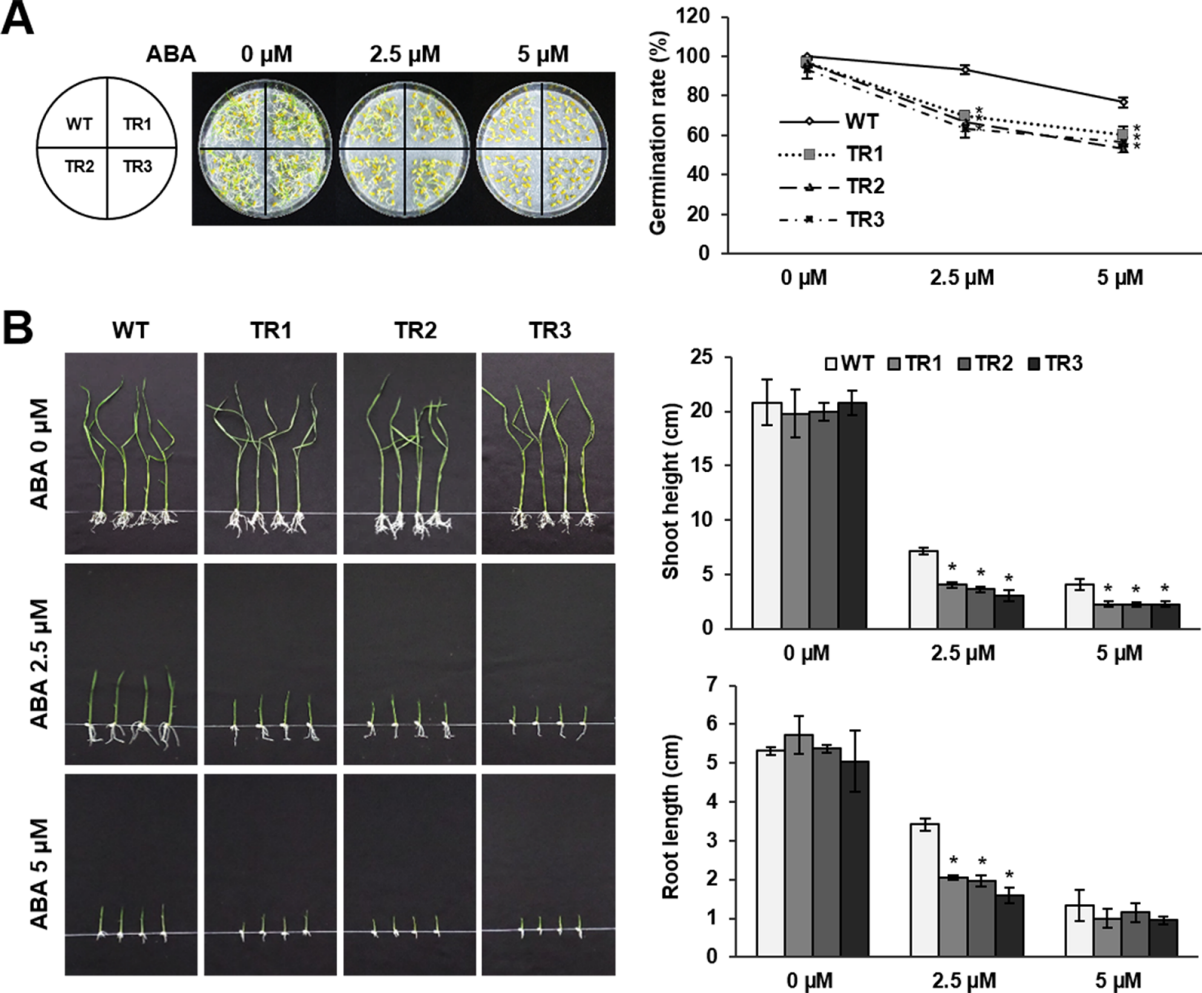
Abscisic acid (ABA) sensitivity in the germination and growth of seeds from *OsASR1*-overexpressing transgenic plants. **(A)** Germination phenotype and percentage of transgenic and WT rice seeds on wet filter paper containing 0, 2.5, or 5 µM ABA 5 d (*n =* 30). **(B)** Shoot and root length of transgenic and WT germinated seedlings grown in 1/2 MS medium containing 0, 2.5, or 5 µM ABA for 10 d (*n =* 10). Asterisks indicate significant differences between treatments determined by Student's *t*-test (*P* < 0.05).

ABA-mediated induction of *OsASR1* expression was confirmed in previous experiments (**Figure 1B**). Therefore, we predicted that *OsASR1* might be involved in ABA signaling. To confirm this hypothesis, seed germination and growth were examined after treating seeds of *OsASR1*-overexpressing transgenic plants with exogenous ABA. Transgenic and WT plants displayed similar germination rates in the absence of exogenous ABA (**Figure 5A**). By contrast, treating seeds with 2.5 and 5 μM ABA significantly reduced germination rates in transgenic plants (66% and 56%, respectively) compared to WT plants (93% and 76%, respectively). These results suggest that *OsASR1*-overexpressing rice plants were more susceptible to ABA during germination than WT plants.

Next, we evaluated the sensitivity of seedling growth to exogenous ABA. In the absence of exogenous ABA, there was no significant difference in seedling growth of transgenic and WT plants (**Figure 5B**). Treatment with 2.5 and 5 μM ABA disrupted the shoot and root growth of transgenic seedlings more strongly than those of WT seedlings. These results showed that *OsASR1*-overexpression increased the susceptibility to exogenous ABA during seed germination and seedling growth, suggesting that *OsASR1* may be a positive regulator of ABA responses.

### *OsASR1* Overexpression Increases Drought Tolerance in Paddy Fields

**Figure 6:**
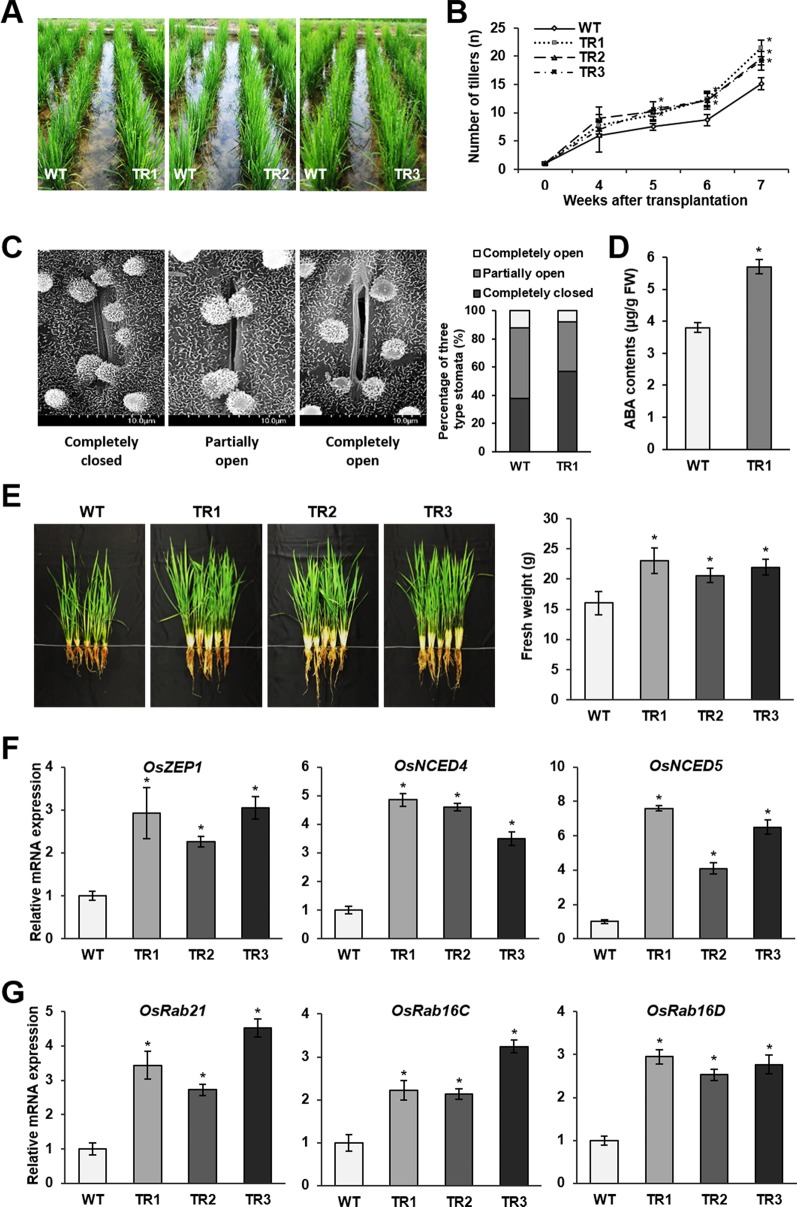
Drought tolerance in paddy fields. **(A)** Phenotypes of *OsASR1*-overexpressing transgenic and WT rice plants 5 weeks after transplanting into the paddy fields. **(B)** Number of tillers on *OsASR1*-overexpressing transgenic and WT rice plants from 4 to 7 weeks after transplanting into the paddy fields. **(C)** Scanning electron microscopy images and the percentages of open, closed, and partially open stomatal apertures in leaves of transgenic and WT plants (*n =* 100 stomata). **(D)** Endogenous ABA contents in leaves of transgenic and WT plants. **(E)** Phenotypes and fresh weight of transgenic and WT plants. Fresh weight represents the value of one plant per each line. **(F)** qRT-PCR analysis of genes involved in ABA biosynthesis (*OsZEP1*, *OsNCED4*, and *OsNCED5*) and **(G)** ABA-responsive genes (*OsRab21*, *OsRab16C*, and *OsRab16D*) from leaves of transgenic and WT plants. Asterisks indicate significant differences between treatments determined by Student's *t*-test (*P* < 0.05). Stomatal image, endogenous ABA content, fresh weight, and qRT-PCR analyses were performed with transgenic and WT plant grown in the paddy fields. Plants were irrigated for 4 weeks and then grown for 4 d without irrigation.

The preceding results prompted us to investigate the drought tolerance of rice plants in paddy fields. Drought conditions were generated by conducting intermittent irrigation starting at 4 weeks after plants were transplanted into the paddy fields. Phenotypic evaluation of *OsASR1*-overexpressing and WT plants revealed a significant difference in the vegetative growth of whole plants (**Figure 6A**). Notably, the number of tillers observed on transgenic plants gradually increased starting at 5 weeks (1 week after the initiation of drought conditions) compared to the number of tillers observed on WT plants (**Figure 6B**). We analyzed the accumulation of reactive oxygen species (ROS) and MDA produced by drought stress. The leaves of WT plants exhibited a purple-blue coloration after staining with an indicator for superoxide anion (O_2_^–^) production, and MDA contents in WT plants were higher than those in transgenic plants (**Supplementary Figure S5**). Next, we evaluated the stomatal apertures in leaves of *OsASR1*-overexpressing and WT plants under drought conditions (without irrigation for 4 d). We used the TR1 transgenic line for this experiment because it had a better overall phenotype under salt and drought stress of the two lines that expressed one copy of the transgene (**Figures 2** and **3**, **Supplementary Table S1**). The percentage of completely closed stomata was higher in transgenic plants (57%) than in WT plants (38%), and the percentage of completely open stomata was 1.5 times lower in transgenic plants than in WT plants (**Figure 6C**). At this point, *OsASR1*-overexpressing plants had greater fresh weight than WT plants (**Figure 6E**).

To confirm whether these results are associated with ABA signaling, we analyzed the ABA contents and expression levels of ABA biosynthetic and ABA-responsive genes in transgenic and WT plants. The results showed that ABA contents were 1.5-fold higher in transgenic plants than in WT plants under drought conditions in paddy fields (**Figure 6D**), which was similar to the results obtained in plants subjected to salt and drought stress in the laboratory (**Figure 4D**). The expression levels of three ABA biosynthetic genes, *OsZEP1*, *OsNCED4*, and *OsNCED5*, were higher in *OsASR1*-overexpressing plants than in WT plants (**Figure 6F**). The expression levels of three ABA-responsive genes, *OsRab21*, *OsRab16C*, and *OsRab16D*, also were higher in transgenic plants than in WT plants subjected to drought conditions in paddy fields (**Figure 6G**). These data further indicated that *OsASR1* might have a major role in regulating the expression of ABA biosynthetic and ABA-responsive genes under drought conditions, thereby regulating stomatal aperture and enhancing drought tolerance.

### *OsASR1* Overexpression Increases Grain Yield Under Drought Conditions in Paddy Fields

**Figure 7:**
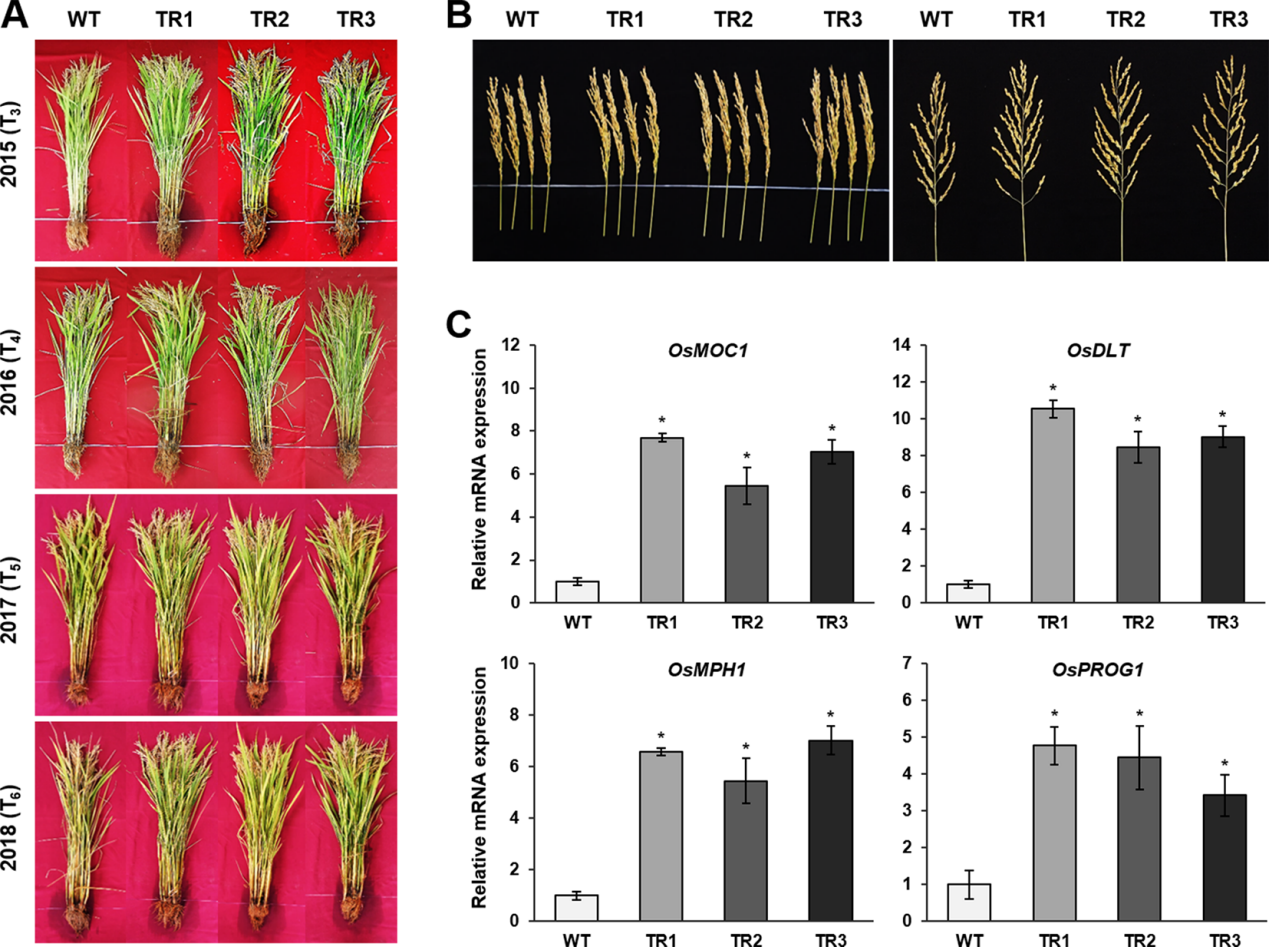
Grain yields from consecutive generations of transgenic plants in paddy fields under drought conditions. **(A)** The phenotype of transgenic plants at the reproductive stage in 2015 (T_3_), 2016 (T_4_), 2017 (T_5_), and 2018 (T_6_). **(B)** Panicle phenotype of transgenic plants at the reproductive stage. **(C)** qRT-PCR analysis of grain yield-related genes from transgenic and WT plants grown in paddy fields for 6 weeks. Asterisks indicate significant differences between treatments determined by Student's *t*-test (*P* < 0.05).

A phenotypic analysis of whole *OsASR1*-overexpressing transgenic and WT plants revealed a major difference in plant growth during the vegetative stage in paddy fields (**Figure 6A**). To evaluate whether this significant physiological difference persists through the reproductive stage, we evaluated the yield components of transgenic plants subjected to drought conditions in paddy fields during four consecutive growing seasons (2015–2018) that had different climatic conditions (**Supplementary Figure S6**). Three independent homozygous lines of the *OsASR1*-overexpressing and WT plants were transplanted into paddy fields and grown to maturity. Transgenic plants produced more tillers than WT plants (**Figure 6B**), which contributed to higher accumulated biomass in transgenic plants than in WT plants under drought conditions in paddy fields (**Figure 7A** and **Table 1**). The total plant weight (g), culm weight (g), root weight (g), number of tillers per plant, and number of panicles per plant during the four growing seasons in *OsASR1*-overexpressing plants were on average 11.4, 18.5, 18.0, 16.8, and 15.8% greater, respectively, than in WT plants (**Table 1**). The higher numbers of panicles and spikelets in *OsASR1*-overexpressing transgenic plants contributed to higher grain yields than those in WT plants (**Figure 7B** and **Table 1**). These results prompted us to analyze the expression patterns of several genes (*OsMOC1*, *OsDLT*, *OsMPH1*, and *OsPROG1*) involved in the regulation of tillering, which in turn determines panicle number, a key component of grain yield (Li et al., 2003; Bolle, 2004; Jin et al., 2008; Tong and Chu, 2009; Zhang et al., 2017). The expression levels of these genes were measured in leaf tissue sampled 6 weeks after transplanting, which is when the number of tillers increased dramatically. Gene expression in transgenic plants was upregulated at least 4.7-fold in transgenic plants compared to WT plants at this stage (**Figure 7C**). These combined results suggest that *OsASR1* overexpression significantly improves grain yield by increasing drought tolerance in rice and increasing the expression of productivity-related genes at the reproductive stage.

**Table 1:** Agronomic traits of *OsASR1*-overexpressing transgenic plants grown in paddy fields under drought conditions in 2015–2018.

**Line**	**Total plant** **weight (g)**	**Culm weight (g)**	**Root weight (g)**	**No. of tillers per plant**	**No. of panicles per plant**	**Total grain yield (g)**	**No. of grains per panicle**	**No. of spikelets per panicle**	**1000-grain weight (g)**
**2015**									
WT	438.0 ± 17.13	280.0 ± 11.55	79.5 ± 7.24	29.4 ± 2.35	27.5 ± 4.23	80.2 ± 5.35	135.9 ± 6.36	12.3 ± 0.12	24.0 ± 0.01
*OsASR1*									
TR1	500.0 ± 54.38 *	340.0 ± 43.58 *	99.6 ± 9.74 *	33.4 ± 5.95 *	32.8 ± 2.63 *	90.9 ± 2.63 *	158.1 ± 15.43 *	13.0 ± 0.23 *	25.9 ± 0.09 *
TR2	510.0 ± 61.18 *	334.0 ± 24.27 *	95.6 ± 5.77 *	36.6 ± 5.45 *	36.6 ± 4.21 *	91.2 ± 4.25 *	148.2 ± 10.36 *	13.4 ± 0.94 *	25.7 ± 0.01 *
TR3	521.6 ± 57.35 *	360.0 ± 27.30 *	99.3 ± 5.53 *	36.2 ± 3.50 *	34.2 ± 7.60 *	91.3 ± 4.51 *	150.2 ± 10.60 *	13.9 ± 0.35 *	25.7 ± 0.04 *
**2016**									
WT	456.0 ± 42.84	270.0 ± 11.20	80.0 ± 3.40	28.4 ± 2.35	29.9 ± 4.23	79.4 ± 5.35	136.5 ± 6.35	13.4 ± 0.12	24.7 ± 0.01
*OsASR1*									
TR1	503.0 ± 53.68 *	335.0 ± 18.23 *	97.1 ± 14.02 *	32.4 ± 2.96 *	31.8 ± 4.14 *	90.2 ± 12.26 *	163.2 ± 10.23 *	14.9 ± 0.84 *	25.4 ± 0.01 *
TR2	509.0 ± 41.83 *	342.8 ± 23.33 *	96.6 ± 5.17 *	34.2 ± 5.77 *	34.2 ± 5.21 *	88.0 ± 11.65 *	164.5 ± 8.25 *	15.0± 0.20 *	25.3 ± 0.04 *
TR3	516.0 ± 45.21 *	340.0 ± 20.22 *	98.6 ± 5.81 *	33.8 ± 3.23 *	33.8 ± 2.23 *	92.1 ± 10.32 *	161.2 ± 11.02 *	15.2 ± 0.65 *	25.7 ± 0.02 *
**2017**									
WT	485.2 ± 23.62	298.5 ± 8.16	83.3 ± 4.47	28.3 ± 2.58	27.1 ± 2.21	73.9 ± 10.95	145.1 ± 4.46	13.2 ± 0.45	25.3 ± 0.04
*OsASR1*									
TR1	538.7 ± 23.93 *	350.6 ± 8.16 *	102.5 ± 15.32 *	33.5 ± 8.10 *	32.0 ± 3.16 *	84.2 ± 10.63 *	163.2 ± 15.43 *	14.2 ± 0.23 *	26.4 ± 0.09 *
TR2	541.3 ± 36.84 *	366.0 ± 16.93 *	103.3 ± 9.89 *	35.8 ± 6.05 *	33.0 ± 4.09 *	84.0 ± 4.21 *	164.5 ± 10.36 *	14.7 ± 0.24 *	25.3 ± 0.01 *
TR3	552.9 ± 35.35 *	355.3 ± 35.53 *	115.7 ± 10.61 *	33.2 ± 6.61 *	35.8 ± 3.70 *	85.7 ± 13.60 *	163.2 ± 10.60 *	14.6 ± 0.35 *	26.2 ± 0.04 *
**2018**									
WT	475.5 ± 19.65	264.0± 16.91	76.0 ± 4.64	28.0 ± 2.39	27.4 ± 2.22	75.6 ± 8.80	146.8 ± 15.32	13.0 ± 0.33	24.5 ± 0.08
*OsASR1*									
TR1	510.5 ± 23.30 *	330.0 ± 14.53 *	93.0 ± 10.00 *	33.6 ± 2.19 *	33.0 ± 0.88 *	86.0 ± 10.31 *	166.5 ± 15.35 *	14.3 ± 0.57 *	25.3 ± 0.03 *
TR2	530.3 ± 20.00 *	345.3 ± 20.00 *	92.3 ± 12.02 *	34.5 ± 0.50 *	33.5 ± 1.50 *	85.5 ± 6.32 *	161.5 ± 20.54 *	15.5 ± 0.70 *	25.2 ± 0.02 *
TR3	542.0 ± 35.47 *	350.1 ± 28.87 *	91.3 ± 10.00 *	33.6 ± 1.45 *	32.5 ± 0.67 *	84.9 ± 5.32 *	163.7 ± 16.54 *	14.5 ± 0.70 *	25.0 ± 0.01 *

Asterisks indicate significant differences between treatments determined by Student’s t-test (P < 0.05).

## Discussion

Increases in agricultural salinity and drought have reduced crop growth and crop yields worldwide. It is crucial for future food security to use biotechnology for the development of new crop varieties that can sustain agricultural productivity under these abiotic stresses. Genes that are responsive to saline and drought conditions have been identified through genetic and transcriptomic analysis of plants that are well-adapted to and even thrive in saline soils or drought (Lenka et al., 2011; Ding et al., 2013). For example, *SNAC1*, *OsLEA3-1*, and *OsMIOX* were identified by comparative transcriptome analysis of a drought-resistant upland rice and a drought-sensitive lowland rice, and they have been shown to be involved in drought tolerance (Hu et al., 2006; Xiao et al., 2007; Duan et al., 2012). Several *OsASR* genes were strongly induced by drought stress in an upland, drought-resistance variety but not in a lowland, drought-sensitive variety (Li et al., 2017). Among them, *OsASR5* overexpression contributed to enhanced drought resistance in lowland rice variety, Nipponbare (*O. sativa* L. ssp. *japonica*). In addition, transcriptome sequencing of salt-tolerant halophytes has resulted in identification of candidate genes involved in salinity tolerance (Chen et al., 2016; Mishra and Tanna, 2017). For example, overexpression of an *ASR* gene from the halophyte *Salicornia brachiata* enhanced salt and drought endurance in the salt-sensitive glycophytes *Arachis hypogea* and *Nicotiana tabacum* (Jha et al., 2012; Tiwari et al., 2015). From these studies, it is reasonable to hypothesize that *OsASR1* is a salt and drought stress-responsive gene in rice. To test this, we constructed transgenic lines of the lowland rice variety, Ilmi that overexpressed *OsASR1*.

In our previous study, we founded that the OsASR1 protein accumulated in response to salt stress in Ilmi rice leaves (Kim et al., 2012). This study further confirmed that endogenous *OsASR1* in rice was strongly upregulated in response to ABA, salt, and drought (**Figure 1B**), similar to that observed for *ASR* expression patterns in other plants (Yang et al., 2005; Hu et al., 2013). Many researchers have suggested that the genes induced in response to abiotic stresses confer abiotic stress tolerance (Yanez et al., 2009; Wang et al., 2016a). The endogenous *OsASR1* expression patterns suggested that it might have a positive role in abiotic stress responses to salt and drought. We generated *OsASR1*-overexpressing transgenic plants to investigate *OsASR1* function under salt or drought-stress conditions in the greenhouse and under drought conditions in paddy fields (**Supplementary Figure S1**).

Salt and drought stresses ultimately induce a state of water deficiency in plants by affecting regulation of stomatal aperture (Munns and Tester, 2008; Lim et al., 2015). Stomatal apertures can regulate plant water status by controlling water loss due to transpiration (Hetherington and Woodward, 2003; Kuromori et al., 2018). Subjecting tomato MYB transcription factor mutants (*ars1*) to salt stress conditions resulted in high Na^+^ accumulation and water loss due to high stomatal conductance and transpiration rate caused by loss of stomatal function (Campos et al., 2016). Other studies on salt-tolerant species of Arabidopsis (Wu et al., 2012) and tomato (Koenig et al., 2013) and drought-tolerant rice Azucena (Price et al., 2002) also demonstrated that control of stomatal aperture size and movement has a crucial role in salt or drought stress tolerance and mitigating water loss under salt and drought stress. We observed that, in greenhouse-grown plants subjected to salt or drought stress, a much greater percentage of the stomata of *OsASR1*-overexpressing plants were completely closed than in the WT plants (**Figure 4A**). As a result, the *OsASR1*-overexpressing plants had lower Na^+^ contents, Na^+^/K^+^ ratios, and water loss than WT plants (**Figures 2C–E** and **3B**) due to significantly reduced stomatal conductance and transpiration rates under salt and drought-stress conditions (**Figures 4B**, **C**).

Stomatal aperture is primarily regulated by ABA (Desikan et al., 2004). Drought conditions increase the synthesis and accumulation of ABA in guard cells, causing stomata to close to conserve water (Lim et al., 2015). Seven genes that respond to drought and regulate stomatal aperture in rice have been identified, and five of them (*SNAC1*, *OsSDIR1*, *hrf1*, *SQS*, and *OsCPK9*) are sensitive to ABA (Hu et al., 2006; Huang et al., 2009; Gao et al., 2011; Zhang et al., 2011; Manavalan et al., 2012; You et al., 2013; Wei et al., 2014). These genes represent elements of a complex ABA regulatory network that is not completely understood (Schroeder et al., 2001; Gampala et al., 2002). In this study, we demonstrated that *OsASR1* expression is rapidly induced by exogenous ABA (**Figure 1B**), and seed germination and growth are hypersensitive to exogenous ABA in *OsASR1*-overexpressing plants (**Figure 5**). These knowledge and results suggest that *OsASR1* may be involved in the ABA signaling pathway. Furthermore, the endogenous ABA contents of *OsASR1*-overexpressing plants under salt and drought-stress conditions were much higher than WT plants by enhancing the expression of ABA biosynthesis genes, *OsZEP1*, *OsNCED4*, and *OsNCED5* (**Figures 4D, E**). Further support for this contention is that in *Vicia faba*, overexpression of *AtNCED3* and *AAO3*, genes involved in ABA biosynthesis, dramatically increased ABA levels in guard cells, apparently causing the observed stomatal closure (Melhorn et al., 2008). Also, drought-stressed transgenic *Arabidopsis* plants overexpressing *AtZEP* had reduced stomatal apertures and presumably less water loss than the control (Park et al., 2008). ABA also activates several stress-responsive genes involved in the biosynthesis of osmolytes and LEA-like proteins, which enhance osmotic stress tolerance in plants and mitigate stress-induced damage (Cutler et al., 2010; Fujita et al., 2011). Overexpression of the ABA biosynthesis gene enhanced drought or salt resistance by elevating ABA and osmolytes in transgenic petunia overexpressing tomato *NCED1* (Estrada-Melo et al., 2015) and transgenic tobacco overexpressing alfalfa *ZEP* (Zhang et al., 2016). Based on these studies, it seems reasonable that *OsASR1*-overexpressing plants with high endogenous ABA levels would also increase the levels of osmolytes, such as proline and soluble sugars (**Figures 2G, H** and **3D, E**). Consequently, we analyzed the expression of three *LEA* family genes: *RAB21*, *RAB16C*, and *RAB16D*, which may be responsive to ABA (Roychoudhury et al., 2007). Also, relevant here is the possible importance of Rab proteins in enhancing plant tolerance to abiotic stress (Mazel et al., 2004; Roychoudhury et al., 2007; El-Esawi and Alayafi, 2019). Overexpression of *AtRabG3e* in plants resulted in activation of several mechanisms that protect against osmotic stresses, such as reduced generation of ROS (Mazel et al., 2004). The *OsRab7* gene enhanced drought in transgenic rice by modulating osmolytes, antioxidants, and abiotic stress-responsive genes expression (Mohamed et al., 2001). In this study, our results showed that the expression of *RAB21*, *RAB16C*, and *RAB16D* genes was significantly higher in *OsASR1*-overexpressing plants than in WT plants under salt and drought stress (**Figure 4F**). From this study with drought- and salt-stressed transgenic plants, we conclude that *OsASR1* resulted in enhanced ABA accumulation, which led to induced stomatal closure, osmolyte accumulation, and enhanced expression of *LEA* genes in the ABA-dependent pathway.

Crops in the field are routinely exposed to combinations of environmental stresses (Anjum et al., 2011), and these variable conditions are often not reproduced in controlled studies of stress responses. Notably, transgenic plants with improved tolerance stress in controlled conditions do not always exhibit this tolerance in the field (Mittler, 2006). Therefore, it is important to study the stress response of rice plants under natural paddy field conditions. To do this, we compared the growth of transgenic rice plants that were drought resistance in the greenhouse (**Figure 4**) to WT plants in the field, where we imposed drought stress with intermittent irrigation. The results in **Figure 6** confirm that *OsASR1*-transgenic plants had drought tolerance not only in greenhouse conditions but also in paddy field conditions. We propose that the better growth of the transgenic plants in the fields was, as in the greenhouse, due to increased endogenous ABA, which induced stomatal closure and upregulated *LEA* genes.

We observed drought tolerance and improved productivity in the transgenic plants in the paddy fields over four consecutive growing seasons (**Figure 7A** and **Table 1**). The increased grain yield was primarily caused by increases in the number of tillers, panicles, and spikelets per panicle (**Figures 6B**, **7B** and **Table 1**). There are many studies on genes directly involved in determining grain yield by tillering and panicle branching (Liang et al., 2013). *MOC1* and *DLT* are members of the plant-specific GRAS family proteins, which function in diverse aspects of plant development and responses to abiotic stress (Bolle, 2004). Studies with mutants have demonstrated that these genes promote the production of rice tillers and panicle branches (Li et al., 2003; Tong and Chu, 2009; Tong et al., 2009). *MYB* and *zinc-finger* genes also influence both plant development and ABA-mediated abiotic stress tolerance (Feller et al., 2011; Baek et al., 2015; Wang et al., 2015). Overexpression of *OsMPH1* (MYB-like transcription factor) and *OsPROG1* (zinc-finger transcription factor) leads to increases in grain yield through regulation of plant height and tillers in rice (Jin et al., 2008; Zhang et al., 2017). Our results showed that these genes related to grain yield (*OsMOC1*, *OsDLT*, *OsMPH1*, and *OsPROG1*) were significantly induced in *OsASR1*-overexpressing plants (**Figure 7C**). These combined results suggest that *OsASR1*-overexpressing plants increased grain yield by improving drought resistance due to efficient stomatal closure and upregulating grain yield regulated-genes.

**Figure 8:**
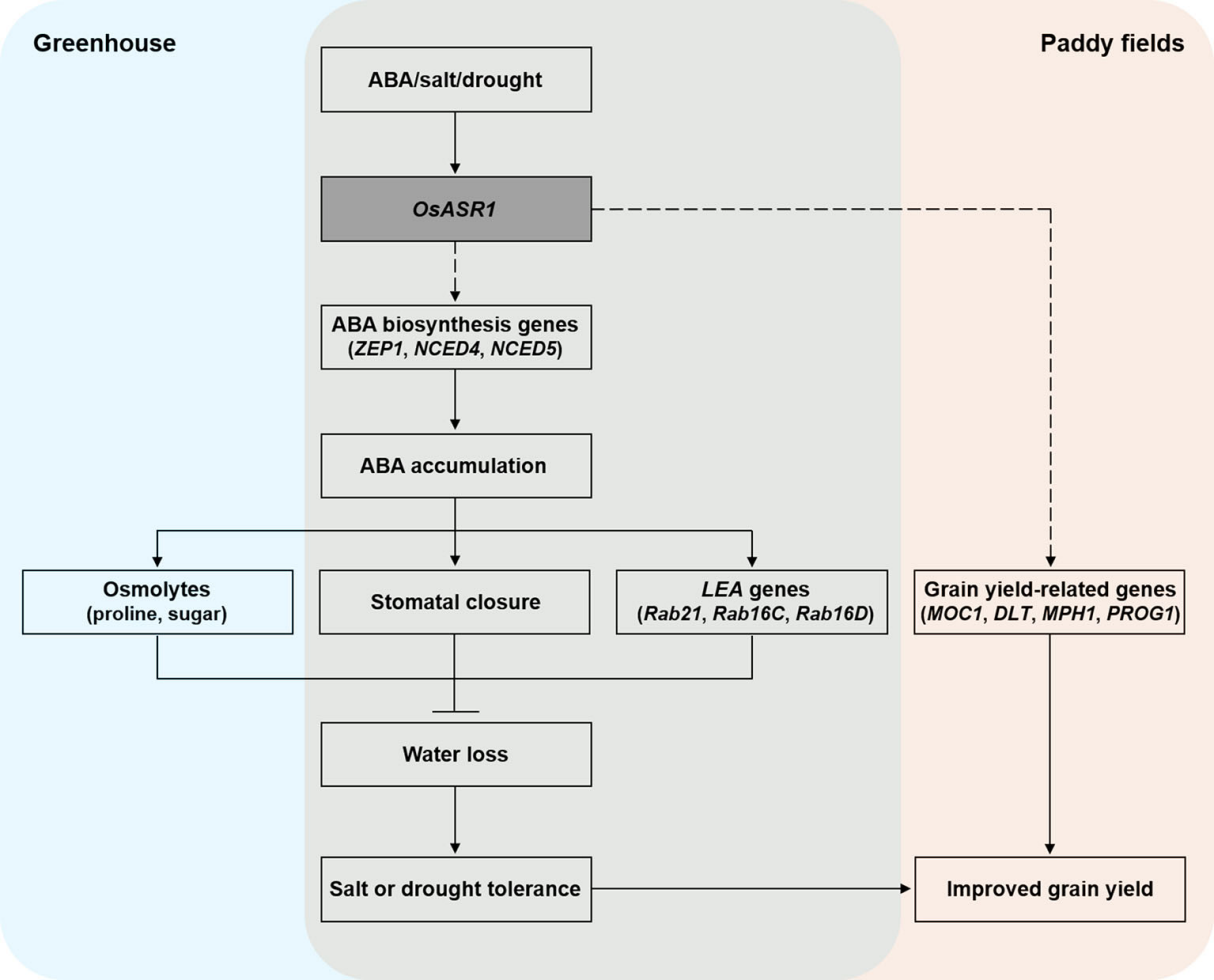
A proposed model describing the function of *OsASR1* in enhancing salt and drought stress tolerance and improving grain yield through modulation of stomatal closure.

Based on our knowledge, we tried to summarize a model to explain the role of *OsASR1* in improving salt and drought stress tolerance and enhancing grain yield through modulation of stomatal closure in plant (**Figure 8**). In conclusion, we demonstrated that *OsASR1* is a positive regulator of ABA accumulation by enhancing the expression of ABA biosynthesis genes. Increased ABA accumulation in *OsASR1*-overexpressing transgenic rice plants leads to enhancing water retention and tolerance to salt and drought stress by modulating osmolytes accumulation, the stomatal closure, and *LEA* genes expression to avoid water loss. *OsASR1* can improve grain yield in rice grown in paddy fields under drought conditions due to improved drought tolerance and upregulation of grain yield-related genes, which indicates that *OsASR1* has a crucial role in drought tolerance and crop improvement. Therefore, this study suggests that *OsASR1* may be an effective transgene for genetic engineering approaches to develop crops that are tolerant to salt or drought stress and have enhanced productivity.

## Data Availability Statement

All datasets for this study are included in the article/**Supplementary Material**.

## Author Contributions

S-IP, Y-SK, and H-SY conceived the research and wrote the article. S-IP performed most of the experiments and analyzed data, J-JK assisted in phenotypic analyses, S-YS constructed the plasmid vector, and H-SY supervised the research.

## Funding

This work was supported by grants from the Next-Generation BioGreen 21 Program (No. PJ01366701), Rural Development Administration, Republic of Korea, and the Basic Science Research Program through the National Research Foundation of Korea (NRF) funded by the Ministry of Education (2018R1D1A3B07049385), Republic of Korea.

## Conflict of Interest

The authors declare that the research was conducted in the absence of any commercial or financial relationships that could be construed as a potential conflict of interest.
