# Supplementary material for: ASR Enhances Environmental Stress Tolerance and Improves Grain Yield by Modulating Stomatal Closure in Rice
Source: Front Plant Sci. 2020 Feb 14;10:1752. doi: 10.3389/fpls.2019.01752 (PMC7033646; doi:10.3389/fpls.2019.01752)
Supplement: Supplementary file 1 [file DataSheet_1.pdf]

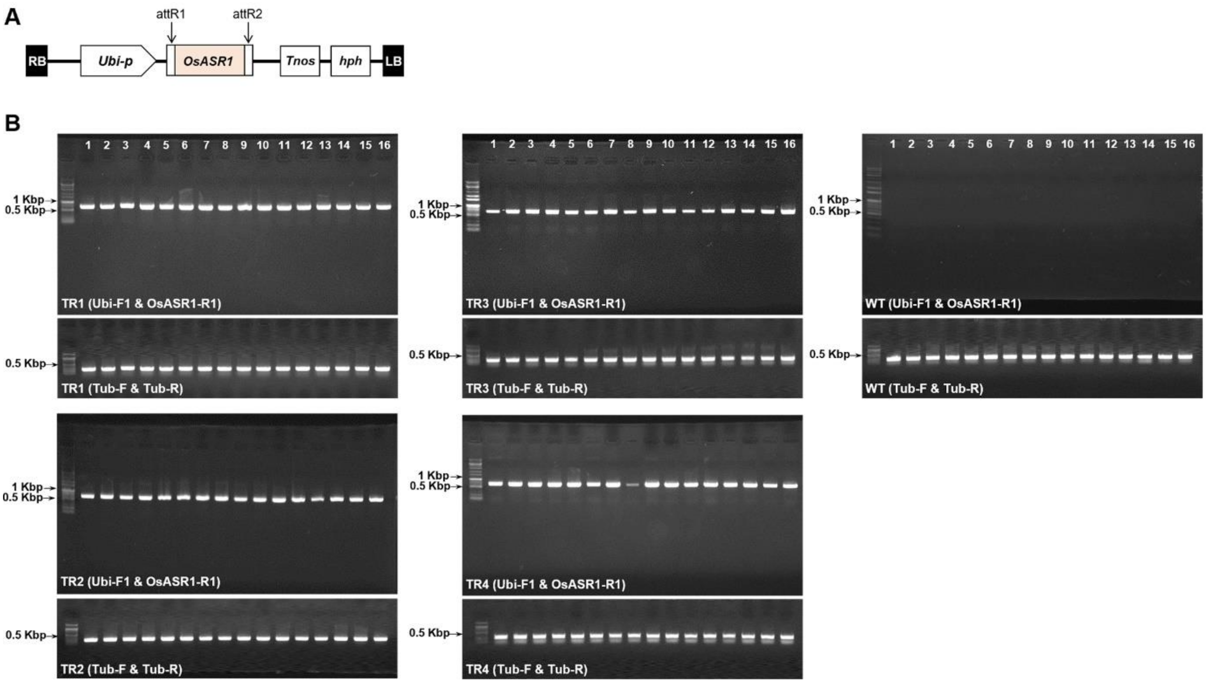

Supplementary Figure S1. Production of *OsASR1*-overexpressing transgenic rice plants. **(A)** Schematic representation of the genomic structure of the *OsASR1* gene including a maize ubiquitin promoter (*Ubi-p*), the *OsASR1* coding regions, and the nos terminator (*Tnos*), which was derived from the nopaline synthase gene. The antibiotic resistance gene, hygromycin (*hgh*), was used as a selection marker. LB, left border; RB, right border; attR1 and attR2, the gateway recombination sites in the *pGA2897* binary vector. **(B)** PCR confirmation of *OsASR1* expression in four independent transgenic plants (TR1, TR2, TR3, and TR4) using primer sets (Ubi-F1, OsASR1-R1, Tub-F, and Tub-R).

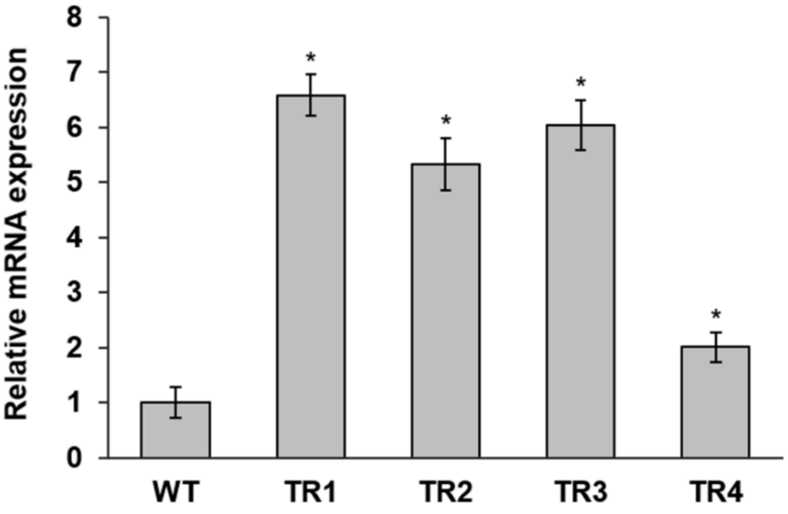

Supplementary Figure S2. Relative expression levels of *OsASR1* in four-week-old *OsASR1*-overexpressing transgenic and WT rice plants.

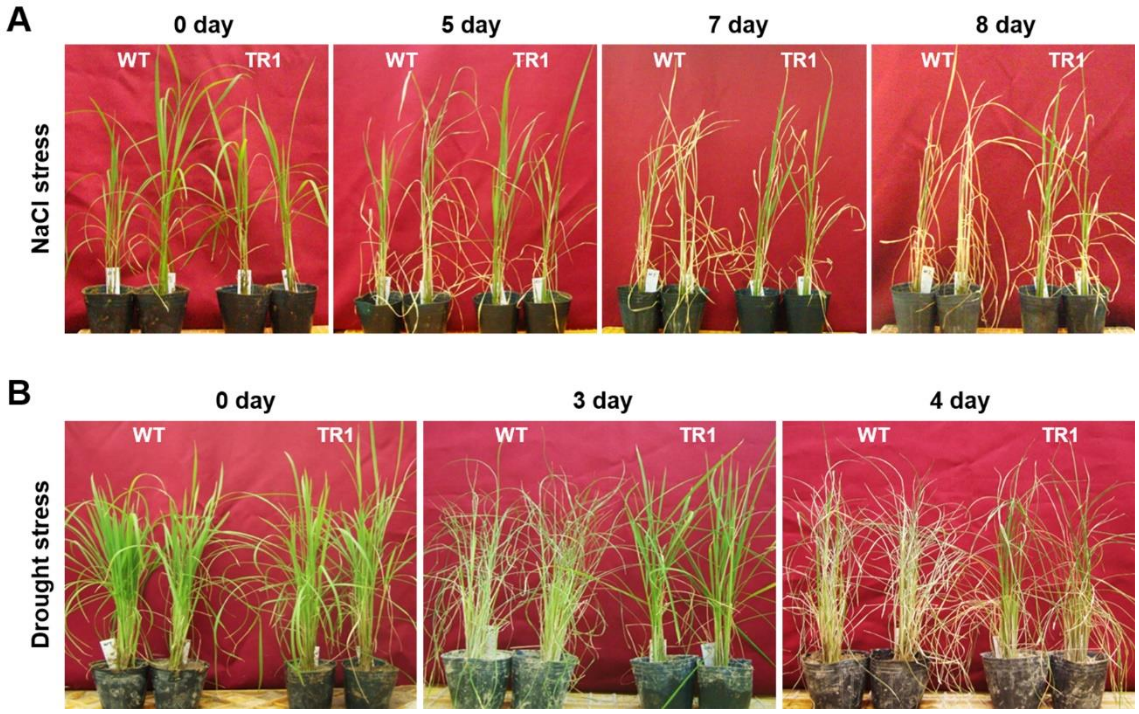

Supplementary Figure S3. Analysis of salt and drought tolerance in *OsASR1*-overexpressing transgenic plants. **(A)** Four-week-old seedlings (*n*=15) after treatment with 200 mM NaCl for 5, 7, and 8 d. **(B)** Four-week-old seedlings (*n*=30) after exposure to non-watered conditions for 3 and 4 d.

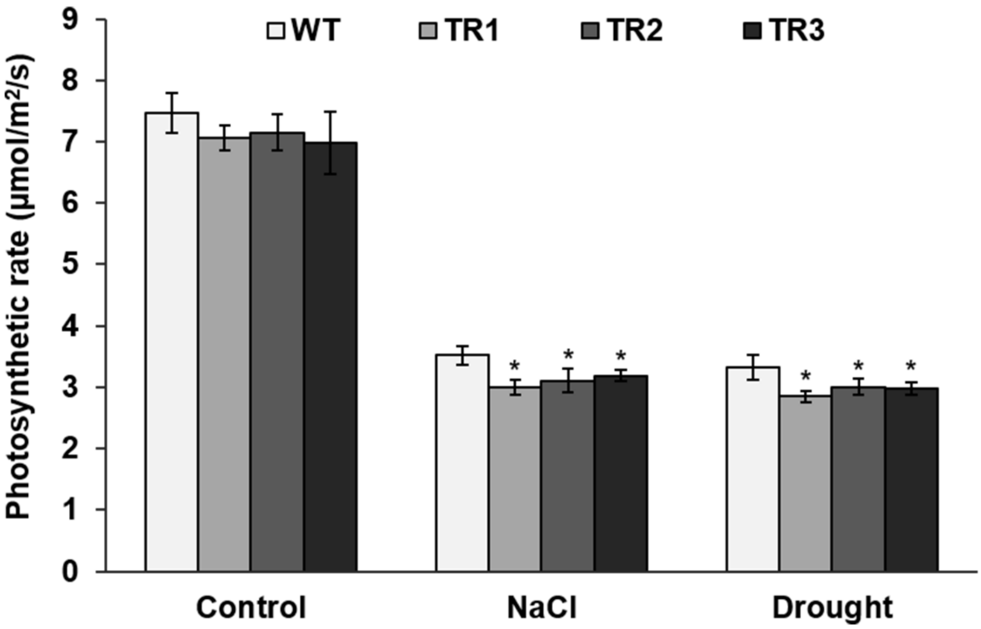

Supplementary Figure S4. Net photosynthetic rate in leaves of WT and transgenic rice plants subjected to salt and drought stress for 5 and 2 d, respectively.

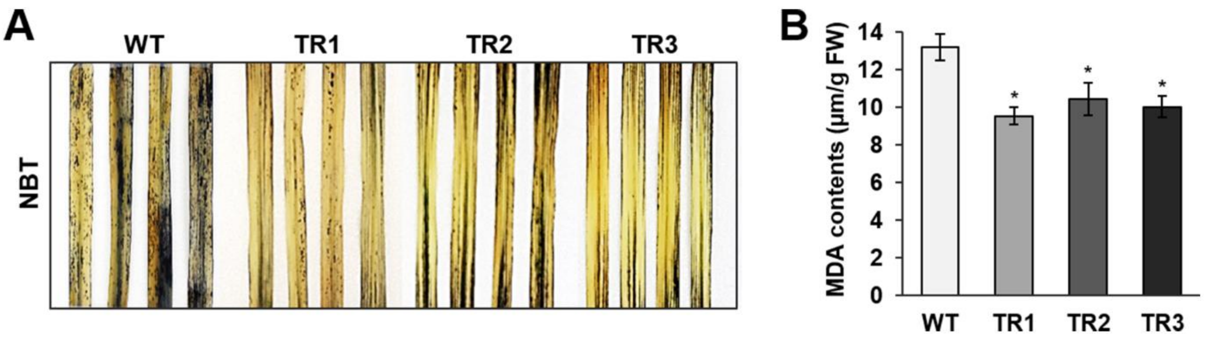

Supplementary Figure S5. Analysis of superoxide ion (O_2^-) and malondialdehyde (MDA) contents in *OsASR1*-overexpressing transgenic plants. **(A)** Phenotype of nitro blue tetrazolium (NBT) staining and **(B)** MDA contents in transgenic and WT plants 7 weeks after transplanting in the paddy fields. Asterisks indicate significant differences between treatments determined by Student's *t*-test ($P<0.05$).

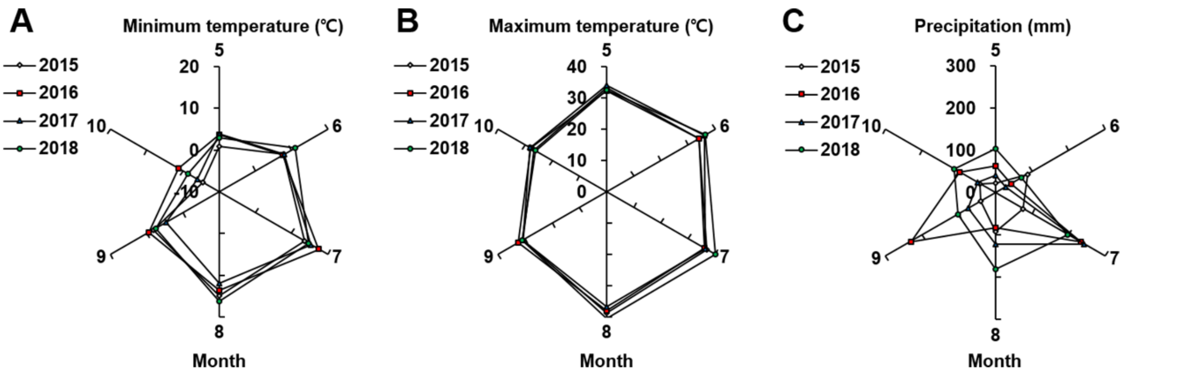

Supplementary Figure S6. Weather conditions during the cultivation periods (May–October) from 2015 to 2018 in the paddy field experiments. **(A)** Average minimum temperature for 6 months. **(B)** Average maximum temperature for 6 months. **(C)** Average monthly precipitation for 6 months.

Supplementary Table S1. Location of gene insertion and copy number through flanking sequence analysis.

Line	Chromosome	Chr_start	Chr_end	Type	Gene ID	Description
TR1	chr12	1167426	1167554	Intergenic	Os12t0124100-01 upstream 2.135kb	Conserved hypothetical protein.
TR2	chr02	8490179	8490353	5'Upstream-1000	Os02t0250300-01 upstream 0.213kb	Bromodomain containing protein.
TR3	chr12	17655452	17655685	Intergenic	Os12t0480300-01 upstream 4.197kb	Hypothetical gene.
	chr02	6677022	6676822	5'Upstream-1000	Os02t0219800-01 upstream 0.096kb	Tetraspanin domain containing protein.

Supplementary Table S2. Primer sequences used in this study.

Oligo name	Oligonucleotide sequences (5´-3´)
OsASR1-FC	CACCTAACTCTGTCTGAAGGAAGCTAAGA
OsASR1-RC	AATTATGGGGAGAAAGTAATTAAGCA
Ubi-F1	CATCTTCATAGTTACGAGTTTAAGATGGAT
OsASR1-F1	AGCACCACCACCTGTTCCAC
OsASR1-R1	ACTAGATCGATCCCGATCGAATGA
Tub-F	GAGTACCCTGCCGCATGAT
Tub-R	GTGGTCAGCTTGAGAGTCCT
OsZEP1-F	TCTGGGACCATTGTCGTTTTT
OsZEP1-R	GCTCAACATCAAAGGCATTCC
OsNCED4-F	GATTGCACGGCACCTTCATT
OsNCED4-R	CTCTGTAATTTGATTTTTCACTGGCTAAT
OsNCED5-F	GGATGGGCTGAACTTCTTCCAG
OsNCED5-R	CAGCACATTCGTGATGAACCCT
OsRab21-F	CACACCACAGCAAGAGCTAAGTG
OsRab21-R	TGGTGCTCCATCCTGCTTAAG
OsRab16C-F	TTCCCGGCCAGCACTAAAT
OsRab16C-R	AAACTGCACGTACATCACGACAT
OsRab16D-F	CGGGTAAACAATAAAGTCGTGATG
OsRab16D-R	GCGCACTTACATACAGTGCTACGT
OsMOC1-F	CCATTGCTGGCCTTATGTGA
OsMOC1-R	CCCCATCATTACCCACCAAA
OsDLT-F	ACGCGGTCGAGGAGTATGG
OsDLT-R	ATCGTCCCTGCAATGGATTG
OsMPH1-F	CATCCTGTCAGCCTCCAGAAG
OsMPH1-R	CCCCTGACGCGTAAATGC
OsPROG1-F	TGAGTTCGGCAAGCAAACG
OsPROG1-R	ATCCTTCCCTCCATTACTCTTCTTC
